# Legal and ethical challenges in assisted reproductive technology practice in Ghana

**DOI:** 10.4314/gmj.v58i1.11

**Published:** 2024-03

**Authors:** Theresa Barnes, Gordon Abakah-Nkrumah, Oboshie Anim-Boamah, Promise E Sefogah

**Affiliations:** 1 School of Nursing and Midwifery, University of Ghana, P. O. Box LG 25, Legon, Accra; 2 University of Ghana Business School, University of Ghana, P.O Box LG 78, Legon, Accra; 3 University of Ghana Medical School, College of Health Sciences, University of Ghana, P. O. Box GP 4236, Accra

**Keywords:** Assisted Reproductive Technology, Ethics, Legal, Ghana

## Abstract

**Objective:**

Infertility remains a global challenge, with assisted reproductive technology (ART) progressively gaining relevance in developing countries, including Ghana. However, associated ethico-legal challenges have not received the needed policy attention. This study explored the legal and ethical challenges of ART practice in Ghana.

**Design:**

The study employed an exploratory phenomenological approach to examine ART in Ghana, focusing on ethics and law governing this practice.

**Participants:**

Respondents were ART practitioners, managers, facility owners, representatives of surrogacy/gamete donor agencies, and regulatory body representatives.

**Methods:**

A semi-structured interview guide was used to collect data.

The in-depth interviews were audiotaped, and responses transcribed for analysis through coding, followed by generation of themes and sub-themes, supported with direct quotes.

**Results:**

It emerged that there are no ethical and legal frameworks for ART practice in Ghana, and this adversely affects ART practice. Ethical challenges identified border on informed consent, clients' privacy and clinical data protection, gamete donation issues, multiple gestations, single parenting, and social and religious issues. The legal challenges identified include the non-existence of a legal regime for regulating ART practice and the absence of a professional body with clear-cut guidelines on ART practice. In the absence of legal and ethical frameworks in Ghana, practitioners intimated they do comply with internationally accepted principles and general ethics in medical practice.

**Conclusion:**

There are no regulations on ART in Ghana. Legal and ethical guidelines are essential to the provision of safe and successful ART practices to protect providers and users. Governmental efforts to regulate Ghana need to be prioritized.

**Funding:**

This study had no external funding support. It was funded privately from researchers' contributions.

## Introduction

Infertility remains a global reproductive health challenge affecting approximately one in six couples and individuals.^1^ Defined as the inability of a couple to achieve conception after a year or more of regular unprotected sexual intercourse,^2^ 10-20% of couples experience infertility with significant regional variations.^3^ In Africa, childbearing is a socially binding duty and couples with infertility suffer major societal stigma.^4^,^5^,^6^ Commonly, women get blamed for the couple's infertility, although male factors may also cause infertility. This results in low self-esteem, social isolation and divorce, with some women suffering physical and emotional abuse from partners.^7^,^26^

Advancements in medical diagnostics and therapeutic technologies in human reproduction have led to the development and use of Assisted Reproductive Technology (ART). This includes in-vitro fertilization (IVF) and embryo transfer, gamete intrafallopian transfer, zygote intrafallopian transfer, tubal embryo transfer, gamete and embryo cryopreservation, oocyte and embryo donation, and gestational surrogacy.^3^,^8^

ART has gained increased relevance in developed countries, with substantial progress being made in developing economies, including Ghana.^9^ ART in developing countries has reportedly been associated with major ethical-legal challenges that have not received the needed attention from policymakers10. Even though ART has been practised over the past four decades, with increasing interest in the field globally^11^,^12^ including developing countries,^13^ none of these developing countries, including Ghana, has established national laws and specific ethical guidelines to regulate ART.^6^ The existing laws and ethical principles that guide general medical practice are inadequate to regulate ART.^14^,^15^

As a result, some related contentious legal and ethical questions continue to remain largely unanswered in most developing countries. Some of these questions have been answered differently across different countries based on socio-cultural beliefs and personal experiences influenced by spiritual backgrounds and personal convictions.^1^,^4^,^6^,^10^,^16^

A previous study observed that many countries, especially in Africa, have no clear-cut legislation on the operations and activities of ART facilities.^17^ Hence, most ART facilities rely on guidelines of the American Society of Reproductive Medicine, the British Human Fertilization and Embryology Authority or the equivalent bodies in France or Germany for guidance.^17^,^18^

Ethical issues in ART are profound, controversial and heavily debated.^5^,^19^,^20^ In the United States of America, the debate has focused on regulation for ART practice, multiple pregnancies, preimplantation genetic testing, gamete donation, privacy, surrogacy, gestational carriers, embryo donation and social inequities.^20^ The focus of ART facilities, however, has been on the clients' financial ability to pay, so that financially incapable families become excluded from accessing ART care and, to some extent, marginalized.^21^ Ethics and laws in ART help clients to easily assess their fertility treatment and simultaneously protect them from exploitation by unscrupulous elements.^20^ The absence of both ethics and laws in developing countries becomes detrimental to the overall success of ART practice.^17^,^20^,^22^ This study adopts the deontological ethical theory of morality as an appropriate ethical theory in examining the concept of ART practice in an environment where ART is still largely in its nascent stages. Admittedly, ethics alone is not enough as there is a need for laws to be enacted to guide ART practice.^17^ Laws in medical practice are concerned with the prerogatives and responsibilities of medical professionals and the rights of the patients.^23^

Data from the Fertility Society of Ghana (FERSOG) confirms that ART practice in Ghana is increasing rapidly. There are about fourteen centres in Ghana, of which nine are registered with the Society. Presently, FERSOG does not have legal authority or responsibility to regulate ART in Ghana. Proper regulation of the industry through appropriate laws and a professional regulator for ART in Ghana has therefore become essential.

This study therefore sought to explore the main legal and ethical challenges pertaining to ART in Ghana, with the view to providing evidence-based data for policymakers, legislators, and other relevant stakeholders to support the call to provide regulation and ethical guidelines for improving ART practice in Ghana and other developing settings.

A conceptual framework was developed based on two theories (Deontology and the New Natural Law Theory) for the study. The theories expound how law and ethics can be deployed to resolve the dilemma faced by both practitioners and clients and to achieve acceptably standardized ART practice in Ghana.

## Methods

This was a multi-centre qualitative study using an exploratory phenomenological approach to explore Assisted Reproductive Technology in Ghana, focusing on ethical and legal challenges in eight out of the fourteen ART facilities in Ghana. The eight facilities were purposively sampled. From these facilities, thirteen respondents who consented to voluntarily participate in the study were recruited in addition to three representatives from regulatory bodies, giving a total of sixteen respondents comprising three Embryologists, six Obstetrician Gynaecologists (4 of whom own ART facilities) and two nurses. Further, one representative from the Nursing and Midwifery Council of Ghana, one representative from the Medical and Dental Council, two respondents from Surrogacy/Donor Agencies, and one representative from the Ministry of Health, Ghana, were selected for the study. These key informants had at least over one year of experience in their field of work related to ART service. For purposes of confidentiality, the names of the ART facilities as well as the names and positions of the clinicians and respondents from the regulatory agencies were anonymized.

The interview guide was designed, pre-tested at a separate facility and modified prior to data collection. The interview questions were formulated based on the literature review to help obtain perspectives on ethical and legal challenges in ART practice in Ghana. The questions in the interview guide included the demographic characteristics of the respondents; their perspectives on ART practice in Ghana; commonly encountered ethical challenges; legal challenges in ART practice in Ghana and how practitioners navigate these challenges; current state of regulation in ART practice and the challenges thereof; recommendations for ethical and legal regulation of ART in Ghana.

Written informed consent was obtained from the selected ART facilities and respondents prior to interviews. Interviews were conducted in the English language, recorded and transcribed verbatim by the principal investigator. The audio-recorded interviews were listened to several times, and the transcripts were read over again to ensure the researcher was familiarized with the data. This was followed by coding and generation of themes and subthemes from the data. These were presented narratively using themes and sub-themes, supported by verbatim quotes from the respondents. Ethical approval was obtained from the Ghana Health Service Ethics Review Committee (GHS-ERC 007/01/21).

## Results

A summary of the study participants is presented in Table 1.

**Table 1:** Categories of stakeholders

Category of Stakeholders	Number of Respondents
**Practitioners: consisting of embryologists, obstetrics, gynaecologists, and nurse**	7
**Facilities/Agency Owners:**	6
**Regulators: representatives from the Ministry of Health, Medical & Dental council, and Nursing and Midwifery council**	3
**Total**	16

**Source:** Field Data, (2020)

The findings were grouped under three thematic areas: ethical challenges, legal challenges pertaining to ART practice, and the effects of ethical and legal challenges on ART services in Ghana.

### Ethical Challenges in ART practices

It emerged from the findings that there are no clear local ethical protocols that are specifically harmonized to guide ART practice in Ghana. All the respondents revealed that there are no ethical guidelines pertaining to ART practice in Ghana. As a result, ART practice is befogged with the ethical challenges of informed consent, clients' privacy, protection of clinical data, gamete donation issues, multiple gestations, single parenting and religious factors.

### Informed Consent

The client is required to give his/her permission and acceptance to undergo the ART service. Unfortunately, there are no specifically developed ethical guidelines to provide content that is specific for purposes of obtaining informed consent in the specialized area of ART service provision. As a result, most practitioners navigate this by using standard informed consent for other general medical or surgical procedures.

*“Any form of service rendered in ART is preceded by the client's informed consent. This is because, the clients cannot be coerced to patronize ART services.” (Embryologist)*

*“…We give our clients all the necessary information on all the relevant ART services in our domain. Thereafter, we furnish them with a consent form to sign before we proceed to start their treatment cycle.” (Embryologist)*

*… because we lack the standardised format of consenting which should be uniform across all centres in Ghana, every centre has some form of consenting they administer to clients or sometimes, a general pre-operative consent form is slightly modified for use. There is the need for national harmonisation of things like this by law” (OBGYN Specialist)*

### Clients' Privacy and Clinical Data Protection

These require absolute adherence to ethics. All the respondents stressed the need to protect clients' privacy and data both in general medical and ART care provision. The respondents posited that clients' privacy and data protection are always respected during the treatment. Clients are counselled on ART, including success probabilities, gamete donation, multiple gestations, and surrogacy. Specifically, the respondents from the surrogacy and gamete donor Agencies emphasized that clients' privacy and data protection are very serious ethical issues when it comes to gamete donation, IVF and surrogacy:

*“…. when it comes to gamete donation, we often make sure our clients' information are protected. Most of our gamete donors are from tertiary institutions and we ensure that their information is protected, likewise the recipients of donor gametes or embryos. We put up systems that ensure that both parties do not know, or come in contact with each other, as this may generate complications in the future. With surrogate mothers, we make sure their information is protected from the general public. To this end, we always isolate these surrogate mothers from the community in which they leave.”*

*“… you know sometimes the gamete recipients wish to see the data and photos of the donors, or even meet them if possible. But in the absence of established guidelines on these situations, we just have to find our own innovative ways of navigating these situations on daily basis. Occasionally if the recipient insists, we ask the donor's permission and share their photo, showing just the face alone.”*

### Gamete Donation Issues

The major ethical concern raised on gamete donation is the potential risk of incest in the future. Presently, there are no laws or ethical provisions specifically relating to the number of times one person can donate gametes in Ghana. As a result, there are no strict ways of checking to ensure donors do not donate too many times. As a result, practitioners use gametes donated without being able to independently confirm how many times the donor has donated previously.

*“… largely, we rely on the prospective donor's word of mouth as to whether she's donated before or not. Sometimes, depending on how you go about it, they would indicate thy have previously donated once or twice previous, without even having disclosed this to their Agencies” (OBGYN)*

*“ … there are no laws on who qualifies to donate and who does not,…. what compensation packages to give the donors, and even surrogate carriers if they carry singleton, if they carry twins, triplets etc, nothing in place for these real-life scenarios in Ghana” (OBGYN)*

*“There is also no established national database or registry of ART cycles or gamete donors to enable easy reference for deciding to use or re-use a donor who previously donated but who may not be forthright with the truth. With appropriate regulation, these would all fall in place to make the practice more transparent for all stakeholders” (OBGYN)*

*“Another thorny issue is the lack of regulation on how much to compensate voluntary donors with. And so, each facility gives what they considered fit as compensation” (Embryologist)*

*“Since most of us also belong to recognized international bodies like the ESHRE and ASRM, we tend to be guided by their guidelines in our line of work”. (OBGYN)*

### Multiple Gestations

Respondents disclosed there are no local ethical provisions on issues relating to multiple gestations. Most clients seeking ART services are desperate, they bear the high costs, and are therefore eager to get favourable results at all costs. And due to their long ‘drought’ of barrenness and infertility, some are desirous of having multiple pregnancies in one cycle. The key ethical drawback in the Ghanaian context is that there are no clear-cut specific ethical guidelines relating to the number of embryos that can be transferred. Counselling by clinicians on the issue of multiple gestation is often related to the associated risk of pregnancy complications and not the ethical reasons.

*… “ after counselling, most clients would ask if they can get twins following the treatment. And you would take time and explain to them the likelihood of twins and the associated potential complications when that happens” (OBGYN)*

*“Once you have three or five babies, the chances of your pregnancy reaching 40 weeks are very slim. At a point, you must be on bed rest, sometimes from 25 to 30 weeks. We must admit you in hospital till you deliver. And the babies that come are very, very small. Hence, we always **advise** based on the risks involved but not based on any ethical guidelines.” (Embryologist)*

### Single Parenting Through Assisted Reproductive Technology

In Ghana, information pertaining to single parenting through ART is still at its nascent stage, with no ethical guidelines on this modality. All the respondents revealed that there are no ethical guidelines on the number of times a woman is entitled under ART, nor is there an ethical framework on how many times a couple can do the same. The embryo recipients and the gamete donors are usually not supposed to interact. Therefore, any dealings pertaining to single parenting are entirely grounded in the parties' prerogative.

### Social and Religious Impacts on ART treatment

A respondent (an Embryologist) revealed that a common ethical issue that often occurs is disagreements among couples on the use of donor sperm. In particular, the husbands would refuse this vehemently and sometimes opt out of the treatment entirely. Another ethical concern is the religious and social impact. Some Christians and Muslims believe fertility and childbirth are gifts from God and hence it is improper to conceive using ART to replicate what has been biologically established by God. However, when the couples are counselled, they tend to readily accept the ART interventions subsequently, and are less adherent to this religious restriction.

*…. the religious perception about ART practice has changed overtime due to formal education, but most Muslims do not want the donor gametes from a third party. They want the donation if at all, to be done by them even though their age or medical conditions may not permit success (Embryologist1)*.

Although issues of consent and religious and cultural challenges persist, with thorough explanation, the clients tend to comprehend the concept before signing up for the ART.

*…. Obviously, any institution or in any practice where you need consent and where there is sensitivity like fertility, there's bound to be the issue of cultural diversity and then people's beliefs. But what I have found generally is that knowledge base and education far outweigh cultural influences. People would think that the Muslims wouldn't accept it, but interestingly, we have lots of Muslim patients who have absolutely no problems with the idea. We have people who are grounded in cultural issues and stuff like that who accept IVF with no problems. So, a lot of it has to do with education. Education is the leveler. Education usually would bring everybody with his own diversity or whatever to the level playing field. And if you're well-educated and you have proper information disseminated to these couples, it's usually easy to outweigh any other influences, even religion. So, I think education and counselling actually help to phase out the cultural or religious issues. But then, we still encounter a few. Even when we encounter a few, we are able to cross that barrier over time. The initial introduction of the topic usually brings in various reactions. Ultimately a lot of people go home and think through and call you back. So, religious and cultural issue may not pose big problem” (ART Practitioner / OBGYN)*.

### Legal Issues

Effective ART practice requires the existence of a legal regime enforced by a professional regulatory body with clear-cut guidelines and judicial awareness on ART practices. All the respondents agreed to the non-existence of laws governing ART practice in Ghana and the lack of adequate judicial awareness of ART.

*“There are no laws regulating ART practice in Ghana, there have been some discussions and consultations with stakeholders some years back by the then minister of health but since then, the issue has not come up again for discussion”* - (MoH Rep)

*“.. so far, a few cases that ended up in court were settled through arbitration or based on other broader legal principles and provisions” (OBGYN)*

*… we need specific laws by our parliament to sanitize the system and guide the practice, this is long overdue. Without the specific **laws, there is** a lot of speculation and falsehood by some persons just to discredit practitioners in ART in Ghana.” (OBGYN)*

### Regulatory Body

There is no legally mandated professional body in Ghana that monitors and regulates ART practice. Although most fertility practitioners belong to professional societies, including the Fertility Society of Ghana (FERSOG) and the Ghana Association of Clinical Embryologists (GACE), there are no clear-cut regulatory guidelines on ART in Ghana.

According to respondents, major ethical and legal issues surrounding ART practice do arise but may not get reported. Hence, there is hardly any national data available on these incidents. Minor disagreements between service providers and service users get resolved through education and counselling. However, all the respondents agreed to the pressing need for country-based laws, ethics regulations and guidelines on ART in Ghana. These will help seal off the regulatory lacuna for opportunists who may exploit vulnerable service users. These provisions may also reduce infiltration by quacks into the practice space.

In the absence of a regulatory framework and legal regime, qualified practitioners have formed associations such as FERSOG and GACE in attempts to harmonize their ART practice and guide and update members on new developments in ART. On ethical issues in ART practice, fundamental clinical/medical ethics such as clients' information privacy, data protection and consent are adhered to. However, when it comes to gamete donation issues, multiple gestations, and single parenting issues, ethical standards are adopted from other jurisdictions.

Additionally, the respondents indicated that ignoring these ethical and legal challenges adversely affects ART service provision, where quack professionals or fraudsters may take advantage of the system to cash in on the clients' desperation and vulnerability. Responses were:

*“… there is really no standardized costing for ART treatment in Ghana, so every centre charges amounts based on their own rule. ART costs anything from $4,000 - $7,000 and sometimes more if you do other more sophisticated stuff like embryo testing, sex selection, etc” (OBGYN)*

*“The laws and regulations are very necessary for the protection of both clients and us (practitioners). It will also help protect the market from imposters, quacks and the infiltration by unlicensed individuals. So, we are pushing for the law and regulatory guidelines” (Embryologist)*.

*“The Ministry of Health has oversight responsibility to ensure that a law is enacted to regulate ART. The Ministry therefore needs to work as expected so that fertility experts can also do their work with appropriate legal backing” (OBGYN)*

*“The shocking thing is that the ministry of health lacks a certain degree of awareness of its role as the one to have oversight and direct policy. It is not something that a lot of people over there are interested in, so it took a while for the ministry to respond when FERSOG even approached and said: come and regulate us. Hence there is the need for laws and regulations which will provide us the needed guidance to operate.” (Embryologist)*

*“It actually took the proactiveness of our society FERSOG to begin the process and the ministry then supported and has taken over in pursuing it. Hopefully, we get to have the regulatory policy in place soon.” (OBGYN)*

Respondents also indicated that the law, when enacted must reflect he socio-cultural context of the country and include contributions from other stakeholders in the community.

*“The establishment of laws, regulations and ethics must reflect the country's socio-cultural context. Legitimacy must involve the community, if the people don't recognize the law as reflecting their values and aspirations, forget it. You can't enforce it. That is why a lot of our laws are not enforced” ( Representative, Medical and Dental Council, Ghana)*

In summary, the respondents acknowledged that ethical and legal challenges arise in their ART practice in Ghana. Therefore, it was proposed that the government, in conjunction with other relevant stakeholders, expedite steps in the enactment of laws, establishment of a regulatory body, and provision of ethical guidelines for ART practice in Ghana.

## Discussion

We have examined the ethical, legal and regulatory challenges faced by ART practice in Ghana. ART services are relatively expensive as clients must pay between $4,000 - $7000 or much more in the cases of surrogacy, making it less affordable to many. Demand for ART services is constrained by the high cost. This agrees with Allahbadia et al.,24, who also reported that ART services are very costly in developing countries partly due to private sector monopoly. However, with increasing cases of infertility and its related issues, the demand for ART services is likely to soar soon. Our study found that there are no laws or regulations governing ART practice in Ghana. Hence, practitioners who provide the service do so by conforming to international standards and general ethics of medical practice. Nonetheless, practitioners stressed the need for the enactment of laws and the establishment of a regulatory body to oversee the activities of ART practice because the continuous absence of laws and ethics in Ghana may put both clients and practitioners in jeopardy in the long run. The absence of laws and regulations may also negatively impact the quality of ART services. Brezina and Zhao observed that due to the rapidly evolving nature of ART, legislation and ethical guidelines are often unable to keep pace and address all the ethical and legal issues that are constantly emerging in the field.^20^ The situation is even worse in developing countries where the concept of ART practice is relatively new,^17^,^20^ coupled with absent legal and regulatory controls, as in the case of Ghana.

From our study, practitioners were not overwhelmed by the aforementioned legal and ethical challenges as they were able to navigate them through education, counselling, client information and signed agreements. Nevertheless, practitioners and all respondents reiterated the need for the government and other relevant authorities to expedite action on enacting laws that will help establish a regulatory body to align the operational framework within the socio-cultural context for ART practice in Ghana. Similarly, results from this current study revealed how the absence of ethical guidelines and legal regimes may result in infiltration by quacks, which will compromise the quality of care, the establishment of unauthorized fertility centres for monetary gains, and the lack of protection of clients and practitioners. These may ultimately affect the quality of the ART service provided.

It was therefore recommended from the findings that, since legal and ethical guidelines are essential for safe and successful ART practice, the government should speed up proceedings on providing a legal and ethical framework for ART practice in Ghana. This call is consistent with those of other researchers who emphasized the need for legal and ethical guidelines in ART practice because of the changing nature of continuous innovations in technology.^25^

### Limitation of the study and direction for future studies

The study has established how legal and ethical challenges adversely influence service delivery in ART practice. However, generalizability is limited because of the number of respondents and the research being restricted to urban Ghana. Furthermore, the client's perspective on ethical-legal issues in ART and their impact on ART service was not covered in this study.

### Implications for research, practice and policy

This work gives an in-depth exposition on the nature of ART practice in Ghana and establishes that there are no laws or ethical guidelines for ART practice in Ghana. This apparent regulatory lacuna creates a potential loophole for unscrupulous and money-driven quacks to take advantage of innocent and desperate individuals and couples in dire need of a child. The study, therefore, creates the platform for key stakeholders and policymakers (including Parliament) to hasten engagements in this regard.

The findings from the study have indicated that, in the absence of laws and ethics in ART in Ghana, the few existing practitioners diligently follow the legal and ethical practices in developed countries and those that govern medical practices in general so that, in the absence of laws and ethics in a particular medical field, general ethics must be upheld by practitioners.

## Conclusion

ARTs are emerging approaches to addressing infertility but are associated with inherent ethical and legal challenges. ART practice in Ghana is still in its embryonic stages and lacks both ethical and legal regulation. These ethical and legal regulatory frameworks are needed to protect clients, agencies, and clinicians and promote sanity in the field of ART in Ghana. Though practitioners abide by internationally accepted principles, they recommended the need for Ghana's own context-appropriate laws and ethical guidelines on ART.

## Figures and Tables

**Figure 1:**
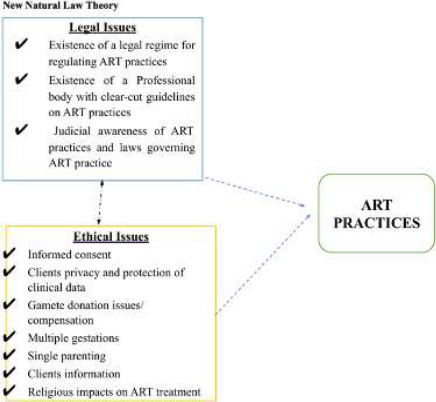
Conceptual Framework on ethics and legal challenges in ART practice *(Source: Developed by Researcher)*

**Table 2:** Sub-themes of ethical and legal isues

THEMES	SUB-THEMES
**Ethical issues in ART practice**	Informed consent Client's privacy, and protection (financial and clinical data) Gamete donation issues Multiple gestations Single parenting Religious impact on ART treatment
**Legal issues in ART practice**	The existence of a legal regime for regulating ART. The existences of a professional body with clear-cut guidelines on ART Judicial awareness **of** ART practices
